# Supplementary material for: Synergistic Binding of bHLH Transcription Factors to the Promoter of the Maize NADP-ME Gene Used in C4 Photosynthesis Is Based on an Ancient Code Found in the Ancestral C3 State
Source: Mol Biol Evol. 2018 Apr 5;35(7):1690–705. doi: 10.1093/molbev/msy060 (PMC5995220; doi:10.1093/molbev/msy060)
Supplement: Supplementary Data [file msy060_supp.zip › Supplementary Figures.pdf]

Supplementary figures

Supplementary FIG. S1.

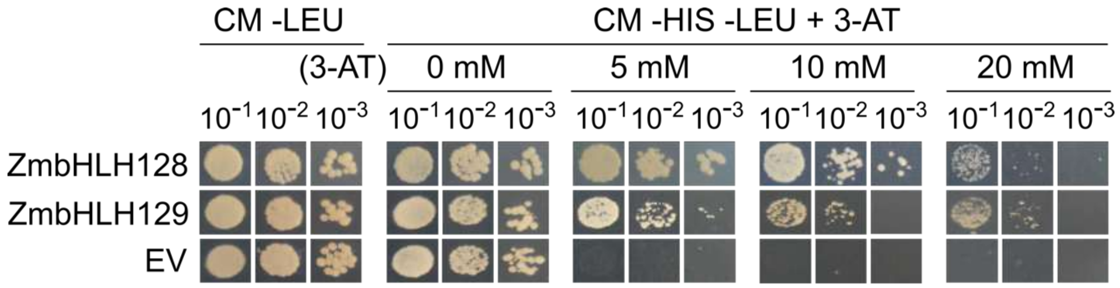

Supplementary FIG. S2.

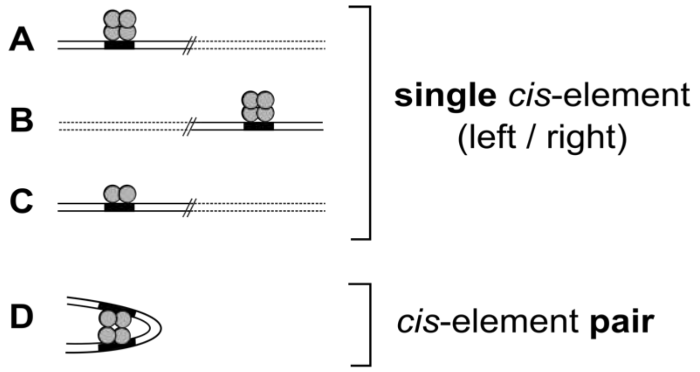

A probe 1
B probes 2, 4
C probes 5, 7, 9, 11
D probes 1+2, 3+4, 5+6, 7+8, 9+10, 11+12

Supplementary FIG. S3.

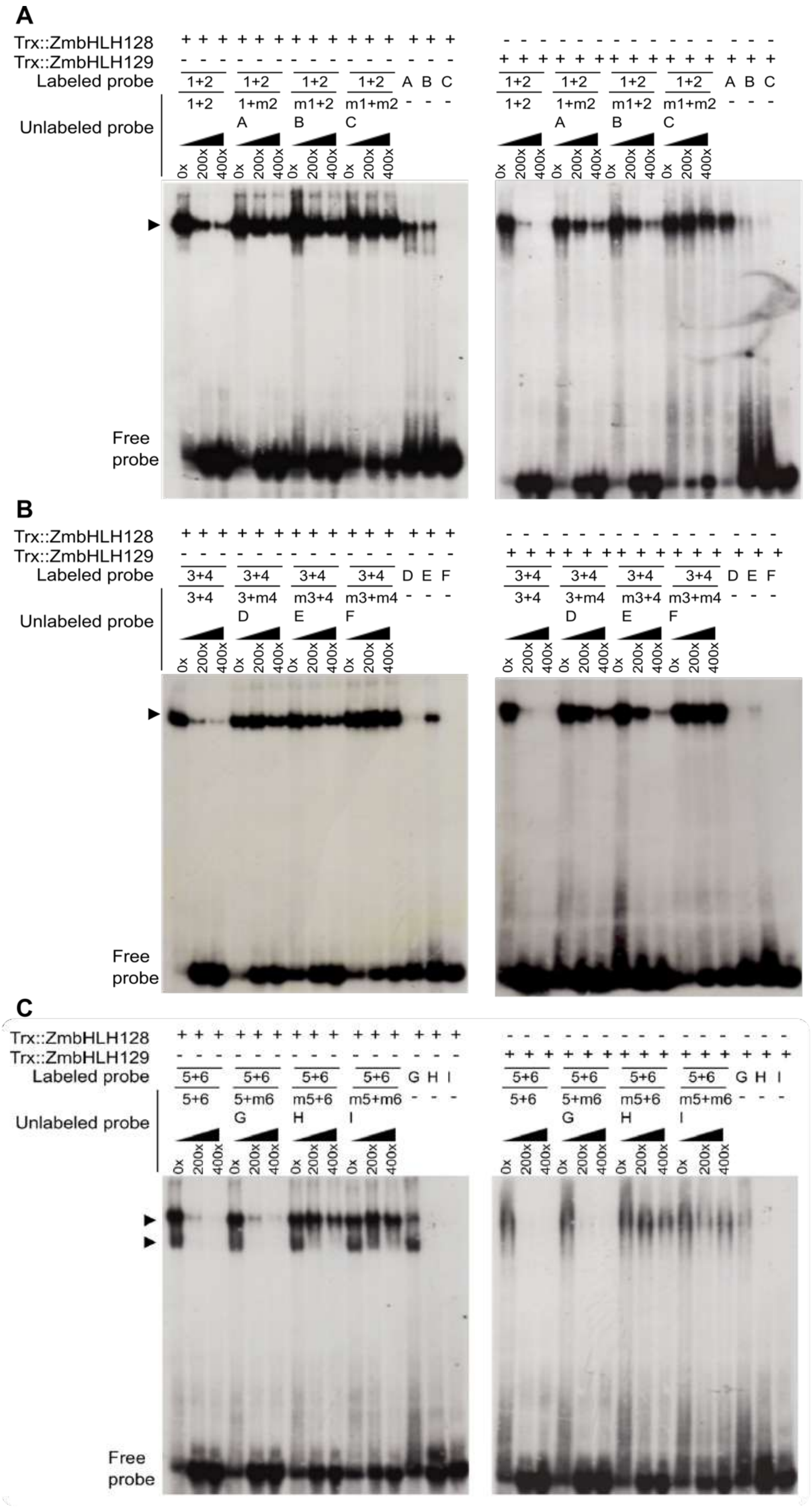

Supplementary FIG. S3

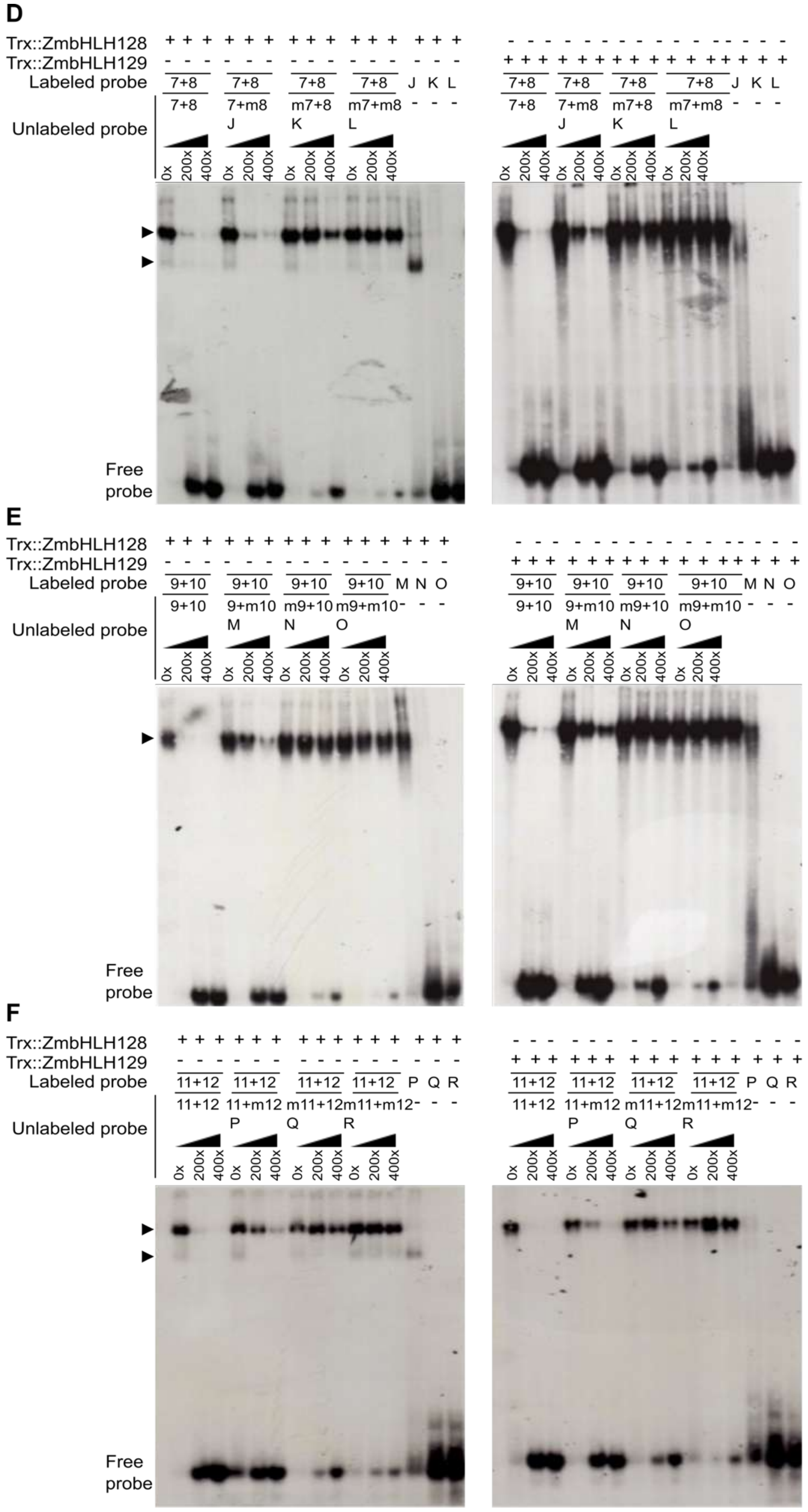

Supplementary FIG. S4.

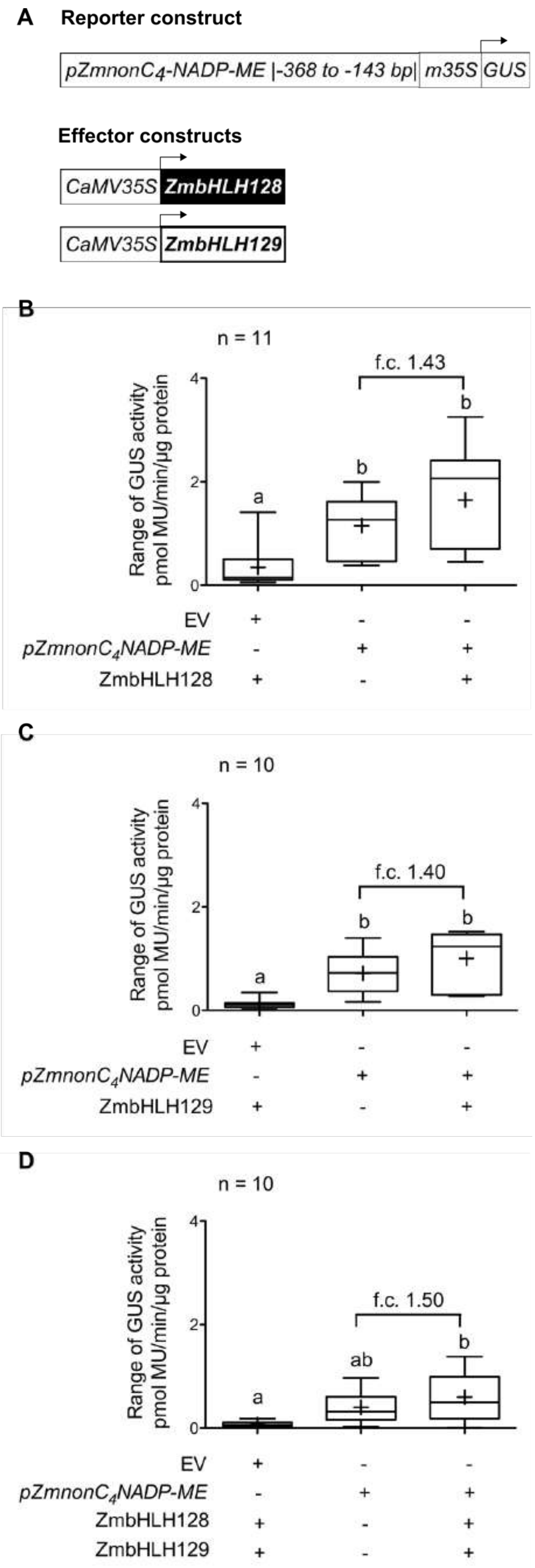

Supplementary FIG. S5.

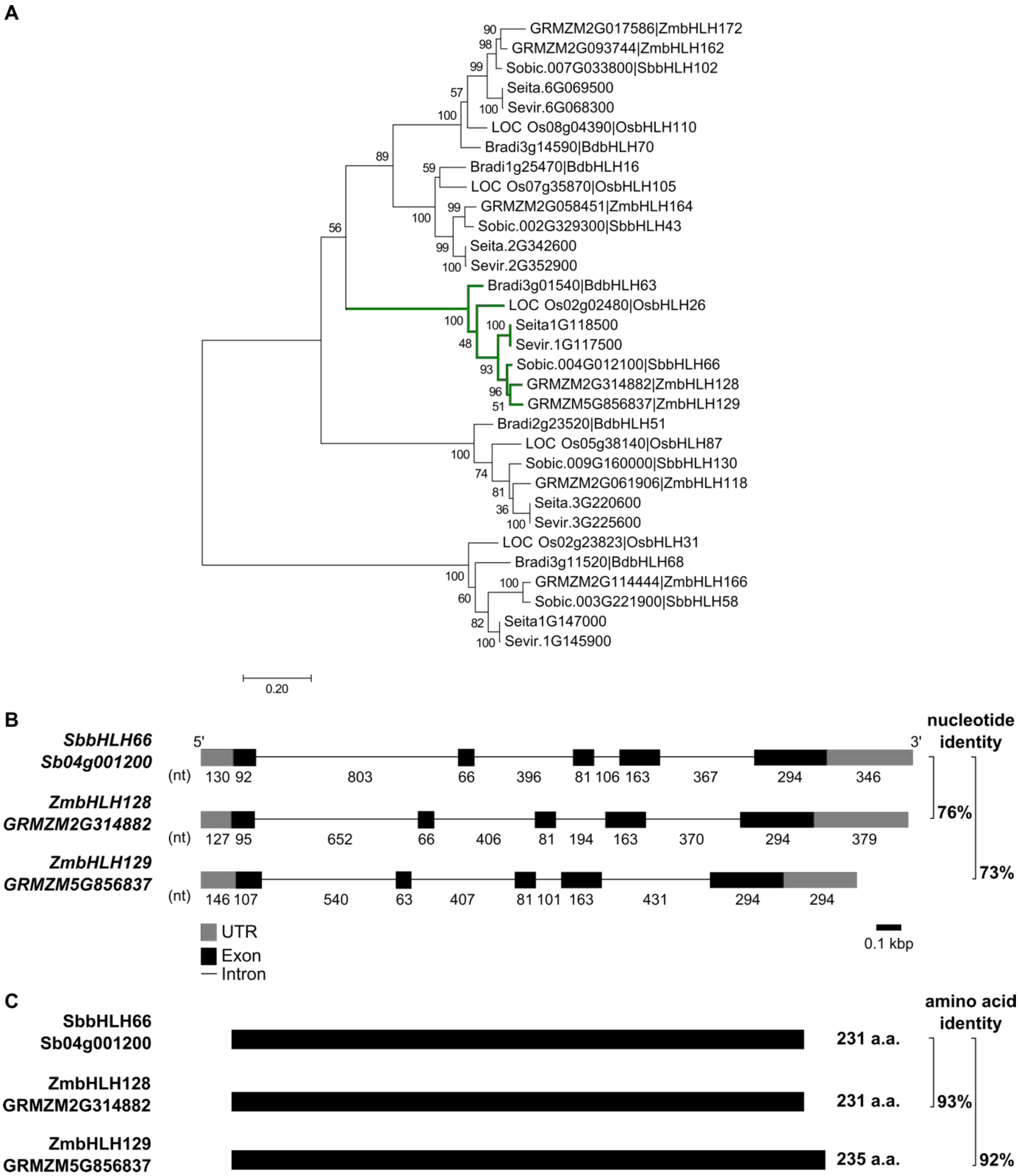

Supplementary figure legends

Supplementary FIG. S1. Analysis of ZmbHLH128 and ZmbHLH129 binding strength to the *ZmC_4-NADP-ME* promoter sequence between -389 to -154 bp. CM -LEU indicates yeast Complete Minimal medium lacking leucine; CM -HIS -LEU + 3-AT indicates yeast Complete Minimal medium lacking histidine and leucine, and supplemented with increasing concentrations of 3-AT: 5, 10 and 20 mM. EV indicates pAD-GAL4-2.1 empty vector (no TF cloned).

Supplementary FIG. S2. Proposed mechanism of ZmbHLH128- or ZmbHLH129-DNA assembly. Black rectangles represent *cis*-elements (probe and *cis*-element sequences are listed in supplementary table S3). Two and four circles represent bHLH homodimers and tetramers, respectively.

Supplementary FIG. S3. ZmbHLH128 and ZmbHLH129 bind specifically to *cis*-elements present in *NADP-ME* promoters. (*A*-*F*) EMSA showing that unlabeled probe competitors in molar excess directly suppress *in vitro* binding of Trx::ZmbHLH128 and Trx::ZmbHLH129 to specific *cis*-elements identified in the following promoters: (*A*) *ZmC_4-NADP-ME*, (*B*) *SbC_4-NADP-ME*, (*C*) *SiC_4-NADP-ME*, (*D*) *ZmnonC_4-NADP-ME*, (*E*) *SbnonC_4-NADP-ME*, and (*F*) *DoC_3-NADP-ME*. 'm' indicates mutant probes carrying double mutation in specific *cis*-elements (probe sequences and mutated *cis*-elements are listed in supplementary table S3). Black triangles represent increasing amounts of unlabeled probe competitors (200x and 400x). Arrowheads indicate uplifted ZmbHLH-DNA probe complexes. Free probe indicates unbound DNA probes.

Supplementary FIG. S4. Analysis of ZmbHLH128 and ZmbHLH129 *trans*-activation activity on the *ZmnonC_4-NADP-ME* promoter. (*A*) Schematic representation of reporter and effector constructs used in transient expression assays in leaves of *Nicotiana benthamiana*. Reporter construct contains *GUS* gene driven by the minimal *CaMV35S* promoter (m35S) fused to the p*ZmnonC_4-NADP-ME* (-368 to -143 bp). Effector constructs contain *ZmbHLH128* or the *ZmbHLH129* CDS driven by the full *CaMV35S* promoter. (*B*-*D*) Box plots (2.5 to 97.5 percentiles) showing GUS activity, expressed in picomoles of the reaction product 4-methylumbelliferone (MU) generated per minute per microgram of protein, in leaves agro-infiltrated with reporter and the following effector constructs: (*B*) ZmbHLH128, (*C*) ZmbHLH129, and (*D*) ZmbHLH128

and ZmbHLH129. Different letters denote differences in experimental data that are statistically significant (One-way ANOVA, Tukey test, $p \leq 0.05$, n = 10-13). EV indicates pGWB3i empty vector (no promoter fragment cloned). Cross inside box plots indicates mean. f.c. indicates fold-change.

Supplementary FIG. S5. SbbHLH66 represents the direct sorghum orthologue and ZmbHLH129 diverged more from the ancestral gene. *(A)* Phylogenetic analysis of *ZmbHLH128* and *ZmbHLH129* closely related *bHLH* CDS sequences of *Zea mays*, *Sorghum bicolor*, *Setaria viridis*, *Setaria italica*, *Oryza sativa*, and *Brachypodium distachyon*. CDS were aligned using MUSCLE and phylogenetic tree inferred by maximum likelihood method (1000 bootstrap pseudoreplicates, node numbers indicate bootstrap values). *(B)* Gene structure of *SbbHLH66*, *ZmbHLH128*, and *ZmbHLH129* orthologs. nt indicates nucleotides. UTR, exon and intron lengths provided from Phytozome. *(C)* Protein alignment of SbbHLH66, ZmbHLH128, and ZmbHLH129 orthologs. a.a. indicates amino acid residues. Nucleotide *(A)* and amino acid *(B)* sequences were aligned using MUSCLE.
